# An interdisciplinary integrated specialized one-stop outpatient clinic for idiopathic intracranial hypertension – a comprehensive assessment of patient satisfaction

**DOI:** 10.1186/s10194-024-01835-x

**Published:** 2024-08-01

**Authors:** Gabriel Bsteh, Stefan Macher, Nik Krajnc, Wolfgang Marik, Martin Michl, Nina Müller, Sina Zaic, Jürgen Harreiter, Klaus Novak, Christian Wöber, Berthold Pemp

**Affiliations:** 1 Department of Neurology, Medical University of Vienna, Waehringer Guertel 18-20, 1090 Vienna, Vienna, Austria; 2 Comprehensive Center for Clinical Neurosciences & Mental Health, Medical University of Vienna, Vienna, Austria; 3 Department of Neuroradiology, Medical University of Vienna, Vienna, Austria; 4 Department of Ophthalmology, Medical University of Vienna, Vienna, Austria; 5 Division of Endocrinology, Department of Internal Medicine, Medical University of Vienna, Vienna, Austria; 6 Department of Neurosurgery, Medical University of Vienna, Vienna, Austria

**Keywords:** Idiopathic intracranial hypertension, Patient satisfaction, Relation, Staff competence, Management, Setting, Facilities, Accessibility, Availability, Outcome, One-stop outpatient clinic

## Abstract

**Background:**

Management of idiopathic intracranial hypertension (IIH) is complex requiring contributions from multiple specialized disciplines. In practice, this creates considerable organizational and communicational challenges. To meet those challenges, we established an interdisciplinary integrated outpatient clinic for IIH with a central coordination and a one-stop concept. Here, we aimed to evaluate effects of this one-stop concept on subjective patient satisfaction and economic outcome in patients with IIH.

**Methods:**

In a retrospective cohort study, we compared the one-stop era with integrated care (IC, 1-JUL-2021 to 31-DEC-2022) to a reference group receiving standard care (SC, 1-JUL-2018 to 31-DEC-2019) regarding subjective patient satisfaction (assessed by the Vienna Patient Inventory). Multivariable binary linear regression models were used to adjust for confounders.

**Results:**

Baseline characteristics of the IC group (*n* = 85) and SC group (*n* = 81) were comparable (female: 90.6% vs. 90.1%; mean age: 33.6 vs. 32.8 years, educational level: ≥9 years of education 60.0% vs. 59.3%; located in Vienna 75.3% vs. 76.5%). Compared to SC, management within IC concept was associated with statistically significantly higher subjective patient satisfaction (beta = 0.93; *p* < 0.001) with the strongest effects observed in satisfaction with treatment accessibility and availability (beta = 2.05; *p* < 0.001). Subgroup analyses of patients with migration background and language barrier consistently indicated stronger effects of IC in these groups.

**Conclusions:**

Interdisciplinary integrated management of IIH statistically significantly and clinically meaningfully improves patient satisfaction – particularly in socioeconomically underprivileged patient groups. Providing structured central coordination to facilitate and improve access to interdisciplinary management provides means to further improve outcome.

**Supplementary Information:**

The online version contains supplementary material available at 10.1186/s10194-024-01835-x.

## Introduction

Idiopathic intracranial hypertension (IIH; formerly also referred to as pseudotumor cerebri or benign intracranial hypertension) is a syndrome of increased intracranial pressure of unknown etiology [1]. Considered rare in the general population, IIH typically occurs in obese women of childbearing age with incidence increasing markedly due to the obesity pandemic [2, 3]. Main health associated risks of IIH include visual field loss and blindness if not treated in time, as well as disabling and often chronic headaches [4, 5].

Treatment of IIH should include a combination of weight loss, pharmacological treatment, and, in severe or refractory cases, invasive neurosurgical intervention [4, 6, 7]. Due to the increasing complexity of managing patients with IIH, international consensus guidelines recommend that IIH care should be provided in specialized centers with access to the necessary resources and therefore recommend interdisciplinary management of IIH [8, 9]. Despite this broad consensus, there are very few descriptions in the literature as to how such inter- or multidisciplinary management should be structured and organized in practice.

We have recently established an interdisciplinary integrated special outpatient clinic for IIH at our center providing a one-stop approach to diagnosis and treatment aiming to improve care and increase patient satisfaction.

Although such one-stop approaches are often promoted as a means of improving care, especially for chronic diseases with complex management, objective data on their outcome is very scarce. To date, there are no data on the explicit effects of specialized one-stop interdisciplinary integrated care for IIH on patient satisfaction.

## Methods

This study was designed as a retrospective cohort study by analyzing the Vienna IIH Database (VIIH) of Department of Neurology, Medical University of Vienna, which is described in detail elsewhere [10]. As of September 30, 2023, the VIIH database contained a cohort of 289 patients with definite IIH according to the modified Friedman criteria [11]. VIIH case reports contain demographic data, details of diagnostic and therapeutic procedures as well as of the course of IIH.

### Study periods

Study periods covered the time from 1-JUL-2021 to 31-DEC-2022 for integrated care (IC) and 1-JUL-2018 to 31-DEC-2019 for standard care (SC). We chose two identical periods to minimize seasonal effects and we excluded the period from 1-JAN-2020 to 30-JUN-2021 to minimize direct and indirect influences of the SARS-CoV-2 pandemic and the measures to combat the pandemic.

### Intervention group: one-stop specialized interdisciplinary integrated care

**Fig. 1:**
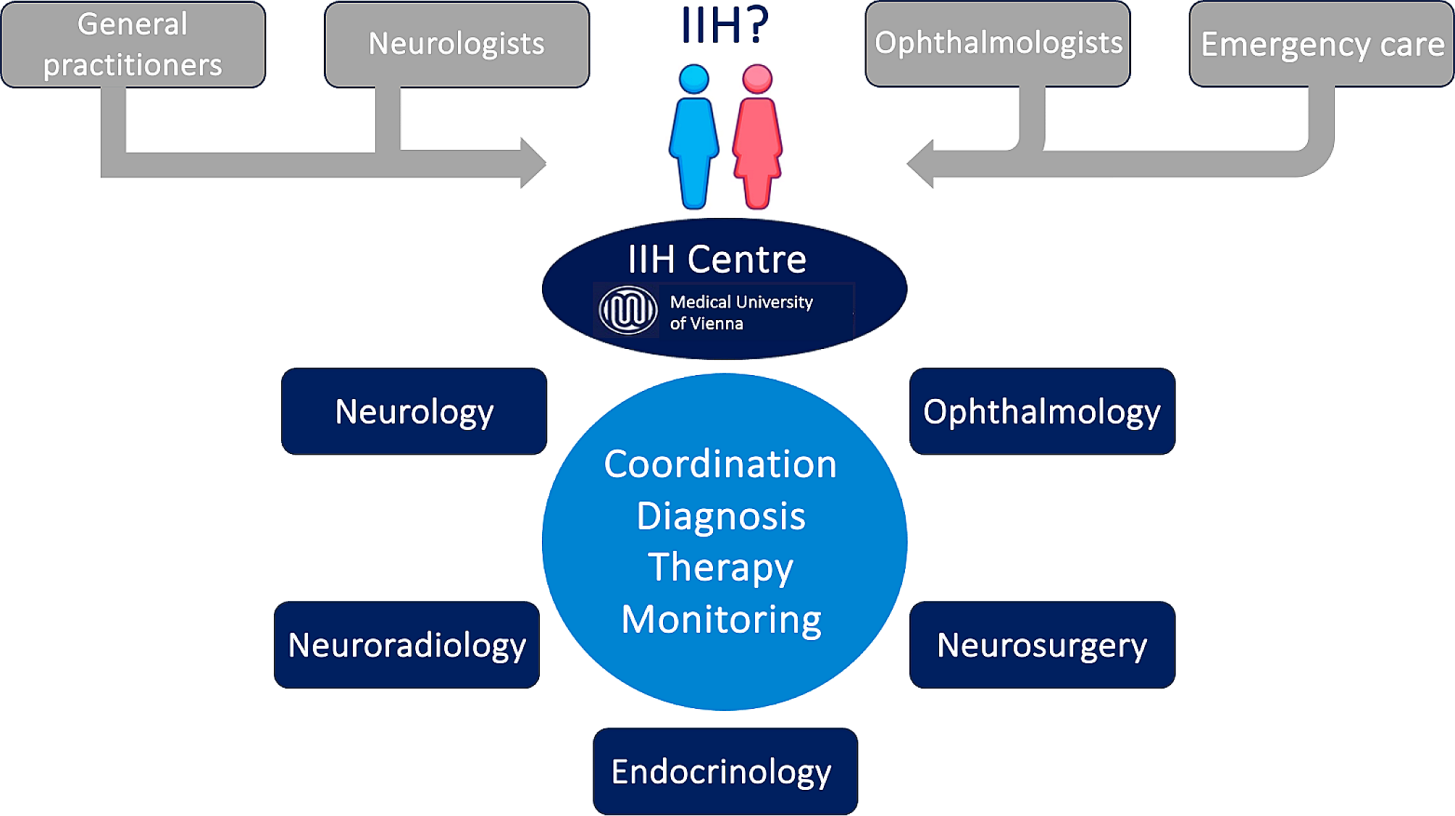
Structural process of the interdisciplinary integrative IIH outpatient clinic in Vienna

The interdisciplinary integrated IIH special outpatient clinic located at the Vienna General Hospital/Medical University of Vienna was established on April 1st, 2021 (Fig. 1). Outpatient care is provided in the outpatient clinics of Departments of Neurology, Neuroophthalmology and Endocrinology, and inpatient care at Department of Neurology and, if necessary, in Department of Neurosurgery. Appointments for examinations and treatment are coordinated through a central coordination by the Department of Neurology to take place at each department at the same day (“one-stop approach”) and communicated to patients in a clear and structured manner in writing. Referrals from specialists in ophthalmology or neurology with a (suspected) diagnosis of IIH are received centrally and reviewed within 2 working days by a specialist from the IIH special outpatient clinic and an appointment for the first examination is made according to urgency. Without referral from an ophthalmologist or neurologist, patients can present themselves independently or on referral from a general practitioner at the general neurology outpatient clinic, from where they can be referred to the IIH special outpatient clinic. Visits are scheduled to last at least 60 min doctor-patient contact (20 min for each neurology, neuroophthalmology and endocrinology) the first presentation and at least 30 min (10 min for each neurology, neuroophthalmology and endocrinology) for check-ups. The results of diagnostic processes and the choice of treatment options for patients of the IIH special outpatient clinic are discussed in a monthly interdisciplinary IIH board meeting chaired by neurology (comprising neuroophthalmology, neuroradiology, neurosurgery and endocrinology) and a joint recommendation is made based on the guidelines of the German Society of Neurology and local standardized operating procedures detailed elsewhere [10, 12]. Necessary prescriptions for drug therapies are requested and issued by the IIH special outpatient clinic and given or sent directly to the patient. For patients with language barriers, a professional interpreter (either in person or via a video interpreting service) is used for all visits.

### Reference group – standard care

SC was assessed in the period before establishment of the IIH special outpatient clinic and required the patients to make appointments for clinical assessments, imaging and other instrumental examinations on their own without centralized coordination or comprehensive use of interpreters. Diagnostic processes and the choice of treatment generally followed the same standardized operating procedures as in the intervention group, apart from the use of glucagon-like-peptide-1-receptor agonists (GLP-1-RA) in patients with IIH and a BMI ≥ 30, which was introduced in March 2022.

### Inclusion and exclusion criteria

We included all patients from the VIIH database with definite IIH according to the modified Friedman criteria and available follow-up of ≥ 6 months. To avoid censored data, patients for whom the period from initial visit to 6-month follow-up was either before the start or after the end of the defined time periods (01-APR-2018 to 30-SEP-2019 or 01-APR-2021 to 30-SEP 2022) were excluded.

### Patient satisfaction assessment

Patient satisfaction was assessed using an adapted version of the Vienna Patient Satisfaction Inventory (WPI), which was given to patients after each outpatient appointment during the respective study periods (1-JUL-2021 to 31-DEC-2022 for IC and 1-JUL-2018 to 31-DEC-2019 for SC) [13]. The WPI maps four main factors of patient satisfaction with treatment: (1) relation and staff competence, (2) management and effectiveness, (3) setting and facilities, and (4) accessibility and availability. Specifically, the adapted version of the WPI comprises 34 items, each of which is answered using a 4-point Likert scale (1 = dissatisfied, 2 = rather dissatisfied, 3 = rather satisfied, 4 = satisfied). The first part of the questionnaire (questions 1–24) contains treatment dimensions that affect all patients, namely overall satisfaction with the treatment, access to treatment, staff-patient relationship including reception at the outpatient clinic and continuity of caregiver, equipment, competence of staff, effectiveness of treatment, and information about and influence on the disease. The second part of the questionnaire (questions 25–34) comprises items on individual services, namely specific treatment interventions (medication, neurology, ophthalmology, weight loss support, and cooperation with relatives), information about medication, and psychosocial support services (satisfaction with help in dealing with authorities and insurance companies, work-related issues, and finances). The addition of the response category “not applicable” to this second part of the questionnaire also makes it possible to indicate which services were not received. The WPI total score was defined as the primary endpoint for patient satisfaction; secondary endpoints comprise the four WPI subscores. In case of more than one available WPI per patient during the respective study period, median scores for each item were used for analyses.

### Covariables

Visual impairment was defined as a visual acuity deviation of ≥ 0.1 logarithm of the minimum angle of resolution (logMAR; determined by Sloan tables at distance after subjective refraction) and/or <-2.0 mean deviation in decibels (dB) in static threshold perimetry determined by the 30 − 2 Swedish Interactive Threshold Algorithm (SITA) [14]. Headache improvement was defined as a ≥ 50% reduction in headache severity (on the numerical rating scale [NRS]) and/or headache frequency (determined by headache days per month) compared to baseline [10].

### Data curation and data analysis

The data relevant to this study were extracted from the VIIH database. The data contained in the VIIH database had already been regularly examined for outliers by two independent auditors (GB and PP). In addition, a random sample of 10% of the recorded patients was analyzed to confirm the quality of the original data collection. In order to further mitigate possible biases in the analysis of retrospective clinical data, a thorough quality control of the extracted data was carried out again for this study, in which the data was examined for outliers and a random sample of 5% of the recorded patients was re-evaluated entirely.

Statistical analyses were performed using R-Statistical Software (version 4.0.0). Univariable group comparisons were carried out as required using the chi-square test, the Fisher exact test, the Mann-Whitney U test or the independent t-test (with Welch correction for unequal standard deviations between the groups). Univariable correlation analyses were calculated using Pearson or Spearman-rho tests, depending on the presence of a normal distribution.

To investigate patient satisfaction, endpoints were initially compared univariabely between IC and SC. Subsequently, multivariable analyses using linear regression models with patient satisfaction endpoints as the dependent variable and group affiliation as the independent variable (IC vs. SC) were performed. Corrected Akaike information criterion (AICc) was used to select the best-fitting model from a predefined set of known relevant covariables (age, gender, educational level [≤ 9 years of schooling vs. high school diploma/ university degree] and place of residence [Vienna vs. outside Vienna]) as well as all other variables associated with the patient satisfaction endpoints with a *p*-value < 0.2 in univariable analyses [15]. We tested all variables for collinearity by variance inflation factor (VIF) and excluded all variables from the regression analyses if the VIF was > 2.0 corresponding to an *R*^*2*^ of 0.50. Predefined subgroup analyses were conducted for patients with a language barrier (defined as German language proficiency ≤ B1) and patients with a first-generation migration background in order to explicitly examine the effects of integrated care on these potentially underserved patient groups. To check for the only systematic difference between IC and SC in standardized operating procedures for treatment, we conducted sensitivity analyses removing patients who received GLP-1-RA. We also conducted sensitivity analyses leaving out patients not newly diagnosed at baseline to check for a potential bias of disease duration. The robustness of all regression models to unidentified confounding factors (bias) was quantified using the Rosenbaum sensitivity test according to Hodges-Lehmann Gamma [16]. Missing values were treated by multiple (20-fold) imputation using the MNAR (Missing not at Random) approach with pooling of estimates according to Rubin’s rules [17]. Significance level was set at a two-sided *p*-value < 0.05.

### Standard protocol approvals, registrations, and patient consents

The study was approved by the ethics committee of the Medical University of Vienna (ethics approval number: 2216/2022). As this is a retrospective study, the ethics committee did not require a written declaration of consent from the study participants.

### Data availability

Data supporting the findings of this study are available from the corresponding author upon reasonable request by a qualified researcher and upon approval by the ethics committee and the data-clearing committee of the Medical University Vienna.

## Results

We included 85 patients in the IC group and 81 in the SC group. Characteristics of both groups are shown in Table 1. There were no statistically significant differences between the groups at baseline, neither in terms of clinical nor demographic aspects.

**Table 1:** Baseline characteristics in integrated and standard care

	One-Stop-Shop (*n* = 85)	Standard care (*n* = 81)	*p*-value
Female^1^	77 (90.6)	73 (90.1)	0.999^4^
Age at diagnosis^2^ (years)	33.6 (9.8)	32.8 (10.3)	0.250^5^
Time from referral to diagnosis^3^ (days)	15 (1–62)	19 (1–82)	0.319^6^
Body Mass Index (BMI)^3^	31.8 (18.2–60.5)	33.0 (17.3–65.6)	0.523^6^
CSF opening pressure (cm H2O)^3^	33 (26–59)	31 (26–63)	0.422^6^
Papilledema grade (Frisen-scale)^3^	3 (0–5)	3 (0–5)	0.872^6^
Disease duration at baseline (months)	0 (0–15)	0 (0–23)	0.832^6^
Visual impairment at baseline^1^	61 (71.8)	56 (69.1)	0.736^4^
Monthly headache days at baseline^3^	18 (0–30)	17 (0–30)	0.644^6^
Chronic headache^1^	47 (55.3)	46 (56.8)	0.877^4^
Headache severity (NRS)^3^	5.5 (0–10)	6.0 (0–10)	0.572^6^
Education level^1^			0.993^4^
≤ 9 years	34 (40.0)	33 (40.7)	
Highschool degree	29 (34.1)	27 (33.3)	
University degree	22 (25.9)	21 (25.9)	
Place of residence^1^			0.851^4^
Vienna	64 (75.3)	62 (76.5)	
Outside Vienna	21 (24.7)	19 (23.5)	
First generation migration background^1^	49 (57.7)	48 (59.3)	0.833^4^
Language barrier (level ≤ B1)	27 (31.8)	28 (34.6)	0.701^4^
Time from diagnosis to treatment initiation^3^ (days)	1 (0–17)	2 (0–29)	0.451^6^

NRS: numerical rating scale. ^1^absolute number (percentage). ^2^mean (standard deviation). ^3^median (range). ^4^calculated with chi-square test. ^5^calculated with t-test for independent groups. ^6^calculated with Mann-Whitney U-test

**Fig. 2:**
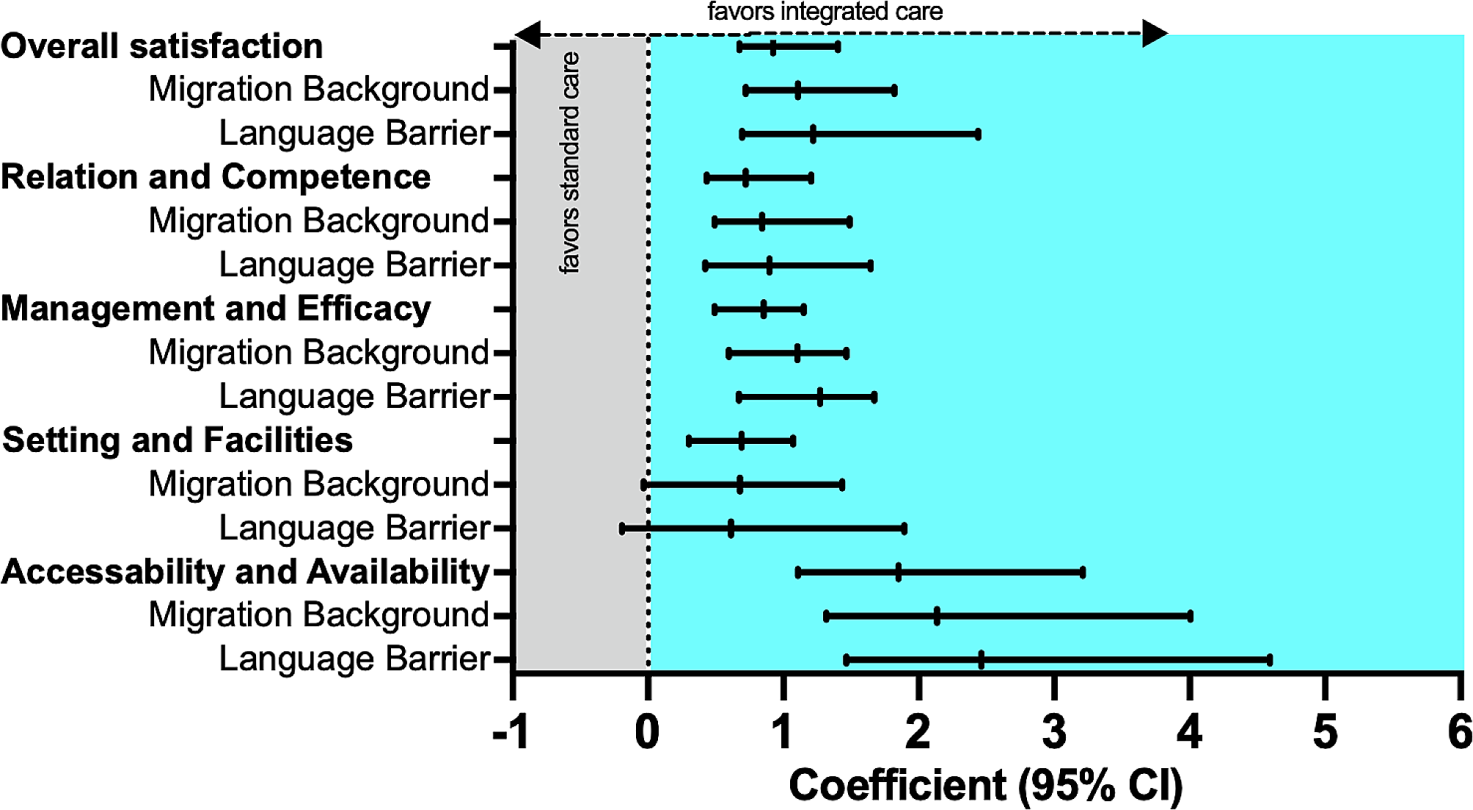
Impact of integrated care on patient satisfaction in the overall cohort and in subgroups with migration background and language barrier

Overall patient satisfaction as measured by mean total WPI score was significantly higher in the IC group than in the SC group (3.30 [SD 0.92] vs. 2.11 [1.01], *p* < 0.001). In the subgroup of patients with a first-generation migration background, mean total WPI score was also significantly higher in the IC group (*n* = 49) compared to SC (*n* = 48) (3.11 [1.04] vs. 1.85 [1.12], *p* < 0.001). In patients with language barrier (German proficiency level ≤ B1), the IC group (*n* = 27) as well displayed a significantly higher mean total WPI score than the SC group (*n* = 28) (3.02 vs. 1.56, *p* < 0.001, Supplemental Table 1). In univariable correlation analyses, higher age (0.119, *p* = 0.002), lower educational level (-0.091, *p* = 0.035), absence of visual impairment (-0.252, *p* < 0.001) and headache improvement (0.231, *p* < 0.001) were correlated with total WPI score. In multivariable analysis, IC was significantly associated with higher overall patient satisfaction with reference to SC (coefficient [β] 0.923, *p* < 0.001, Table 2). The subgroup analyses showed an even stronger association between IC and overall patient satisfaction in patients with migration background (β = 1.108, *p* < 0.001) and with language barrier (β = 1.219, *p* < 0.001, Fig. 2). Higher age (β = 0.112, *p* = 0.011), absence of visual impairment (β=-1.433, *p* < 0.001) and headache improvement (β = 1.198, *p* < 0.001) were independently associated with higher overall patient satisfaction (Table 2).

**Table 2:** Multivariate analysis of the influence of care in interdisciplinary integrated specialized one-stop-shop on patient satisfaction

	Overall Patient Satisfaction		
	β^a^	95% CI	*p*-value
**Integrated care** (vs. reference of standard care)	0.923	0.677–1.402	< 0.001
Age (per 5 years)	0.112	0.005–0.213	0.011
Visual impairment	-1.433	-2.292– -0.911	< 0.001
Headache improvement	1.198	0.874–2.073	< 0.001
	**Patient Satisfaction with** **Relation and Competence**		
**Integrated care** (vs. reference of standard care)	0.722	0.435–1.206	< 0.001
Age (per 5 years)	0.173	0.062–0.289	0.003
Female	0.098	-0.125–0.223	0.362
Visual impairment	-1.522	-2.292 – -1.010	< 0.001
Headache improvement	1.244	0.796–2.003	< 0.001
	**Patient Satisfaction with** **Management and Effectiveness**		
**Integrated care** (vs. reference of standard care)	0.856	0.494–1.149	< 0.001
Age (per 5 years)	0.161	0.041–0.245	0.012
Higher educational level^1^	0.107	-0.085–0.242	0.314
Visual impairment	-1.678	-2.371 – -1.184	< 0.001
Headache improvement	1.373	0.861–2.144	< 0.001
	**Patient Satisfaction with** **Setting and Facilities**		
**Integrated care** (vs. reference of standard care)	0.692	0.302–1.072	< 0.001
Age (per 5 years)	0.121	0.062–0.289	0.003
Female	0.099	-0.104–0.211	0.521
Higher educational level^1^	0.128	-0.027–0.281	0.096
	**Patient Satisfaction with** **Accessibility and availability**		
**Integrated care** (vs. reference of standard care)	1.852	1.107–3.214	< 0.001
Age (per 5 years)	0.169	0.041–0.255	0.010
Higher educational level^1^	0.187	-1.023–1.197	0.783
Place of residence^2^	0.144	-0.186–0.509	0.323

^a^calculated using linear regression models with WPI scores as the dependent variable and group affiliation as the independent variable (integrated care vs. standard care). Positive values indicate a positive association with patient satisfaction. Corrected Akaike information criterion (AICc) was used to select the best-fitting model from known relevant covariates and other variables that were associated with the respective outcome measure with a *p*-value < 0.2 in univariate analyses

^1^high school diploma/ university degree referenced against ≤ 9 years of schooling

^2^resident in Vienna referenced against residence outside of Vienna

Compared to SC, the IC group reported a much higher satisfaction with treatment accessibility and availability (3.59 [0.93] vs. 1.97 [0.87], *p* < 0.001), while also treatment relationship and staff competence (3.45 [0.81] vs. 2.92 [0.87], *p* < 0.001), management and effectiveness (3.22 [0.98] vs. 2.76 [0.92], *p* = 0.002), setting and facilities (2.82 [1.02] vs. 2.45 [0.98], *p* = 0.018) scores were higher but with lesser effect size. Patient satisfaction was also significantly higher in the subgroups of patients with migration background and language barrier in all aspects of patient satisfaction except for setting and facilities (Supplemental Table 1).

Multivariable models revealed a strong association of IC with satisfaction with accessibility and availability (β = 1.852, *p* < 0.001) after adjusting for covariables. Satisfaction with relation and competence (β = 0.722, *p* < 0.001), management and effectiveness (β = 0.856, *p* < 0.001), and setting and facilities (β = 0.692, *p* < 0.001) were also significantly associated with IC, but observed effect sizes were lower compared to accessibility and availability (see Table 2). These associations persisted in the subgroups with migration background and language barrier with slightly stronger effect sizes except for satisfaction with setting and facilities (Fig. 2). Higher age was associated with higher patient satisfaction in all WPI subscores, while both absence of visual impairment and headache improvement independently influenced satisfaction with relation and competence as well as with management and effectiveness (Table 2).

Sensitivity analyses removing patients who received GLP-1-RA (*n* = 24) did not indicate a relevant impact on WPI overall score or WPI subscores. Also, leaving out patients not newly diagnosed at baseline did not significantly change the results. In the study cohort, the proportion of patients not newly diagnosed was similar in the IC group (34.1%, 29/85) and in the SC group (35.8%, 29/81, *p* = 0.871), translating to a similar median disease duration (i.e. time from diagnosis to baseline) of 0 months in both cohorts (*p* = 0.832).

## Discussion

Aiming to describe the effects of one-stop specialized interdisciplinary integrated care for IIH, we found that the integrated one-stop concept was associated with significantly higher subjective patient satisfaction with the strongest effects observed in satisfaction with treatment accessibility and availability, where satisfaction scores improved by more than 1.5 points on the WPI scale ranging from 1 to 4 corresponding to a relative improvement of around 80%. Importantly, subgroup analyses of patients with migration background and language barrier consistently indicated stronger effects of integrated care in these socioeconomically underprivileged groups.

In the literature, there is only one comprehensive description of an inter- or multidisciplinary organizational structure for IIH patients, which is established at the Danish Headache Center in Copenhagen [7]. There are several descriptions of multidisciplinary treatment protocols for IIH, which unanimously advocate the involvement of various specialist disciplines rather than care provided by a single discipline [9, 18–23]. Some protocols are limited to neurology, (neuro)ophthalmology and neurosurgery to identify patients whose visual function is acutely at risk [9, 21, 22]. Others recommend the additional involvement of secondary disciplines or health care professions to address other relevant aspects of IIH, e.g. nutritional counseling and physiotherapy to support weight loss or concomitant psychological and/or psychiatric care to treat patients’ comorbidities such as depression or eating disorders [7, 8, 19, 23, 24]. An interdisciplinary one-stop structure for IIH, such as the Vienna Interdisciplinary Integrated Specialized Outpatient Clinic for IIH, has not yet been described in the field of IIH. Although inter-/multidisciplinary management of IIH is generally recommended, there are no data on the explicit effects of inter-/multidisciplinary structures of IIH care on patient satisfaction and economic aspects.

Patient satisfaction refers to the subjective assessment of medical services or treatments from the patients’ perspective and is therefore an essential parameter for the evaluation of healthcare facilities as a quality indicator in addition to objective clinical outcome [25]. Patient satisfaction comprises various factors (e.g. perceived quality of medical care, communication, or treatment outcome) relating to the perception of “treatment experience”, and thus, needs to be distinguished from the much broader concept of quality of life [25].

We are not aware of any studies evaluating multi-/interdisciplinary care on patient satisfaction in IIH. In general, there is only one study dealing with patient satisfaction of patients with IIH, reporting a very high level of satisfaction among 49 IIH patients with a virtual group consultation offered during the SARS-CoV-2 pandemic to maintain care despite access restrictions [26]. In the present study, patients in standard care were mostly in the medium satisfaction range (between “rather satisfied” and “rather dissatisfied”), whereas the subscores for treatment relationship and accessibility/availability were between “rather dissatisfied” to “dissatisfied”. By contrast, in the one-stop concept average scores for overall satisfaction and 3 out of 4 subscore were moved into the range of “rather satisfied” to “satisfied”, in particular satisfaction with accessibility/availability the one-stop-shop group scored 81% higher. We believe that the main reason for these improvements is the assumption of central appointment coordination in the integrated one-stop approach, as patients in standard care had to attend numerous appointments at various outpatient clinics resulting in a high organizational effort [9]. These improvements withstood correction for potential demographic and clinical confounders in all aspects of patient satisfaction. Of note, we found that higher age was consistently associated with higher patient satisfaction in all categories, while parameters of objective clinical outcome (visual impairment, headache improvement) were expectedly identified as independent predictors of patient satisfaction with treatment management, effectiveness, and staff competence. Associations between gender and education level with aspects of patient satisfaction found in the univariable analyses, did not hold in the multivariable models. The literature is inconsistent on the role of these influencing variables on patient satisfaction in general, so that it may be assumed that there is at least no significant influence of these variables on patient satisfaction in IIH [27, 28].

In the absence of comparable studies in IIH, it is interesting to look at other chronic diseases requiring complex management involving different disciplines. Diabetes mellitus, a chronic disease with a similar risk profile to IIH also benefitting significantly from weight reduction as a therapeutic approach, represents a suitable comparator. Here, a project of integrated, multidisciplinary one-stop approach in the Australian region of Canberra comprising care by a general practitioner, risk assessment, point-of-care laboratory tests, diabetes counseling and podiatry showed a significant improvement in subjective patient satisfaction with care (91% vs. 64% were satisfied or very satisfied) [29]. The main motives cited were significantly increased comfort, promotion of self-management and improved doctor-patient relationships. Unfortunately, no data were available in the present study that would have allowed an exploration of the motives and motivations for the improved perception of patient satisfaction.

However, in our study it was striking that patient satisfaction in the one-stop group was very similar between the overall cohort and the subgroups with migration background and language barrier, whereas these groups reported significantly lower satisfaction scores in standard care than the overall cohort, particularly with regard to accessibility and availability. Standard care therefore appears to disadvantage patients with a migration background and/or language barrier, and the concept of the integrated one-stop concept appears to be suitable for better integrating socioeconomically disadvantaged patient groups. One example of the positive impact of a specialized one-stop concept on disadvantaged patients can be found in the literature: a multidisciplinary program of comprehensive health care for transgender and gender non-conforming adults in the state of Indiana that provides primary care and medical services tailored to the needs of the target population as a one-stop approach, reported consistently very high patient satisfaction [30]. Of course, it must be critically noted here that this was a particularly selected patient group with specific needs that often cannot be adequately covered in other areas of healthcare and is often confronted with discrimination or other negative experiences in personal interactions. This can artificially increase subjective patient satisfaction in a specialized facility, regardless of the actual quality of treatment.

### Limitations

The retrospective design of the study entails various limitations. The lack of randomization may induce several biases, e.g. a selection bias in the sense of an unbalanced selection of patients in a treatment group. However, this is mitigated by the VIIH database, which includes most IIH patients from our geographical area, and the very unselective inclusion criteria [10, 31]. Comparing patients from different time periods could theoretically lead to a systematic bias of the mean shift (Will-Rogers phenomenon), e.g. due to changes in the diagnostic and treatment processes or an immortality-of-time bias [31, 32]. This is particularly relevant because the SARS-CoV-2 pandemic and the measures to combat the pandemic lie between the investigated period of standard care and that of the intervention group. However, the comparison period for standard care was chosen to minimize the direct and indirect influences of the SARS-CoV-2 pandemic and the measures to combat the pandemic. However, it is possible that patient perception and behavior regarding use of medical services may have changed as a result. Still, Rosenbaum sensitivity tests with Hodges-Lehmann Gamma indicated robustness to bias by unidentified confounders [16].

In conclusion, the present study conducted in a representative and large (considering the rarity of IIH) sample of pwIIH shows that one-stop interdisciplinary integrated care independently improves patient satisfaction – particularly in socioeconomically underprivileged patient groups with migration background and/or language barrier.

Providing structured central coordination to facilitate and improve access to interdisciplinary management provides means to further improve outcome. This is deemed especially relevant, as over 90% of pwIIH currently do not have access to inter-/multidisciplinary care [33]. Our data can be leveraged in the interaction with stakeholders and decision-makers to ensure that IIH patients are provided with the best possible care in the most efficient way.

### Electronic supplementary material

Below is the link to the electronic supplementary material.

Supplementary Material 1

Supplementary Material 2

## Data Availability

Data supporting the findings of this study are available from the corresponding author upon reasonable request by a qualified researcher and upon approval by the ethics committee and the data-clearing committee of the Medical University Vienna.
