# Supplementary material for: An interdisciplinary integrated specialized one-stop outpatient clinic for idiopathic intracranial hypertension – a comprehensive assessment of patient satisfaction
Source: J Headache Pain. 2024 Aug 1;25(1):127. doi: 10.1186/s10194-024-01835-x (PMC11295347; doi:10.1186/s10194-024-01835-x)
Supplement: Supplementary file 2 — Supplementary Material 2 [file 10194_2024_1835_MOESM2_ESM.docx]

**Supplemental Table 1. Subgroup analyses for patients with migration background and language barrier of patient satisfaction with integrated care for IIH and standard care.**

|  | **Migration background** | | | **Language barrier** | | |
| --- | --- | --- | --- | --- | --- | --- |
|  | **One-Stop-Shop (n=49)** | **Standard care (n=48)** | **p-value^2^** | **One-Stop-Shop (n=27)** | **Standard care (n=28)** | **p-value^2^** |
| **Overall patient satisfaction ^1^** | 3.11 (1.04) | 1.85 (1.12) | <0.001 | 3.02 (1.30) | 1.56 (1.43) | <0.001 |
| **Relation and staff competence** | 3.62 (0.92) | 3.12 (0.97) | 0.011 | 3.55 (1.02) | 2.87 (1.03) | 0.017 |
| **Management and Effectiveness** | 3.17 (1.08) | 2.42 (1.05) | <0.001 | 3.14 (1.18) | 2.15 (1.28) | 0.005 |
| **Setting and Facilities** | 3.04 (1.02) | 2.56 (0.98) | 0.058 | 3.10 (1.09) | 2.69 (1.03) | 0.158 |
| **Accessibility and Availability** | 3.41 (1.04) | 1.82 (1.10) | <0.001 | 3.37 (1.22) | 1.79 (1.20) | <0.001 |

Determined by WPI (Vienna Patient Inventory) overall score and subscores.

^1^mean values and standard deviation. ^2^calculated by independent t-test.
